# What are the benefits of cultivating self-compassion in adults with low back pain? A systematic review

**DOI:** 10.3389/fpsyg.2023.1270287

**Published:** 2023-11-06

**Authors:** Kellen Greff Ballejos, Prisla Ücker Calvetti, Bruno Luis Schaab, Caroline Tozzi Reppold

**Affiliations:** ^1^Psychological Assessment Laboratory, Department of Rehabilitation Sciences, Federal University of Health Sciences of Porto Alegre (UFCSPA), Porto Alegre, Brazil; ^2^Psychological Assessment Laboratory, Health Sciences Program, Federal University of Health Sciences of Porto Alegre (UFCSPA), Porto Alegre, Brazil

**Keywords:** self-compassion, low back pain, Lumbago, compassion, meditation

## Abstract

**Introduction:**

Low back pain is one of the most prevalent public health problems in the world, generating psychosocial impacts on quality of life and a high demand for medical care. Self-compassion may be beneficial for low back pain control, however, studies in the area are scarce. Therefore, this systematic review aimed to investigate the benefits of self-compassion-related interventions on low back pain and mental health in adults.

**Methods:**

The review protocol was registered in PROSPERO and the method was performed according to the PRISMA guidelines. Searches were conducted using the keywords “self-compassion” and “low back pain” in Portuguese, English, and Spanish in the following databases: PubMed, LILACS, SciELO, PePSIC, PsycInfo, Embase, Scopus, Web of Science, and Cochrane. Additional searches were also conducted through the references of the included studies.

**Results:**

Thirty-three articles were identified and analyzed by two independent reviewers using Rayyan. Four of these studies were included. RoB 2 was used to assess the risk of bias of each study. The main findings suggest that self-compassion-related interventions demonstrate benefits in the treatment of low back pain, as well as reduction in pain intensity, psychological stress, and improvement of pain acceptance.

**Discussion:**

However, these positive data must be analyzed carefully, as only two studies presented a low risk of bias. Despite growing interest in this field, more research self-compassion-related interventions for low back pain are suggested, since biopsychosocial aspects associated with low back pain can impact the outcome of treatment.

**Systematic review registration:**

https://www.crd.york.ac.uk/prospero/, identifier (CRD42022376341).

## 1. Introduction

The cause of chronic low back pain is multidimensional, and its origin involves several factors, including physical, cognitive, psychological, and psychosocial aspects (Malta et al., 2017). In this sense, low back pain has become one of the most prevalent public health issues in the world, generating impacts on the quality of life of individuals affected by this condition and on society through a high demand for medical care (Shipton, 2018; Levenig et al., 2020; Riley et al., 2020; Ünal et al., 2020) and physiotherapeutic care (Pirovano et al., 2023). According to the World Health Organization (WHO) (2003), low back pain affects 80% of the world’s population at some point in their lives. It is the third cause of disability retirement and one of the main causes of absence from work for more than 7 days, being a disease with great impact on productivity and economy. For these reasons, it is among the top 10 causes of medical consultations (Bassols et al., 2003; Refshauge and Maher, 2008).

As described by International Association for the Study of Pain (2019), chronic pain is an unpleasant sensory and emotional experience, based on a biopsychosocial model. On the other hand, low back pain is located above the gluteal fold and below the twelfth rib and is considered chronic in a period equal to or greater than 3 months (de Souza et al., 2016). Chronic low back pain (CLBP) is related to biopsychosocial aspects that interfere with the individual’s quality of life, such as functional disability (Garbi et al., 2014; de Souza et al., 2016; Cargnin et al., 2019; Desconsi et al., 2019), absenteeism, early retirement (Garbi et al., 2014; de Souza et al., 2016), sick leave, depression, suffering (Cargnin et al., 2019), fear (Cargnin et al., 2019; Desconsi et al., 2019), stress, anxiety (Malta et al., 2022), and high treatment costs, with social and financial impacts (Garbi et al., 2014; de Souza et al., 2016).

From the perspective of the biopsychosocial model of treatment, pain should be observed integrally, as it involves subjective beliefs (Desconsi et al., 2019), such as self-perception (Fraga et al., 2019), through which the individual may self-evaluate in a critical and negative way (Pereira and Lourenço, 2012). Thus, non-pharmacological interventions, such as those based on self-compassion, may contribute to pain management and improved mental health. Therefore, the use of self-compassionate attitudes has been increasingly studied to manage subjective negative emotions involved in pain (Neff, 2003a,b, 2011; Nonnenmacher and Pureza, 2019).

Self-compassion pertains to an individual’s capacity to address their own suffering and failures with the intention of alleviating them, much like what we would do for a dear friend (Neff, 2003a,b; Neff and Germer, 2013). Additionally, self-compassion entails treating oneself with care, compassion, and kindness. It is noteworthy that there is a positive association between self-compassion and mental health related to chronic pain. Furthermore, self-compassion may reduce chronic pain (Luo et al., 2020; Vasconcelos et al., 2020).

Despite this, self-compassion is recent in the West, appearing in the literature and in clinical psychology less than 2 decades ago (Gilbert and Procter, 2006; Jazaieri et al., 2013; Neff and Germer, 2013). Self-compassion has Buddhist origins (Greater Good, 2004) and involves self-centered compassionate attributes, allowing self-acceptance in the face of human imperfection, minimizing isolation and self-criticism (Neff, 2003a,b, 2011).

The self-compassion comprises three aspects: (a) kindness and understanding—the individual tends to be kind and understanding toward himself or herself (i.e., less self-critical); (b) sense of common humanity—acceptance and recognition of flaws and defects without isolation; and (c) mindfulness—ability to keep the mind stick in present, aware, and focused on the environment through acceptance (Neff, 2003a,b, 2011; Savieto et al., 2019). These aspects can be measured through the self-compassion scale, which assess the scores of the three components of self-compassion (Neff et al., 2019).

Usually, self-compassion is addressed through two main therapeutic approaches: Compassion-Focused Therapy (Gilbert, 2014) and Mindful Self-Compassion (Germer and Neff, 2019). Despite structural differences, both approaches aim to develop self-compassionate skills (Ferrari et al., 2019). Self-compassion-related interventions encompass exercises that can address the dimensions of self-kindness, common humanity, and mindfulness either individually or in combination, depending on the specific technique (Neff, 2003a,b; Neff and Germer, 2013). For example, practices like loving-kindness meditation simultaneously teach patients how to cultivate both self-kindness and mindfulness (Neff, 2003a,b; Neff and Germer, 2013). There are numerous other techniques available for nurturing self-compassion, including composing self-compassionate letters, visualizing a compassionate self, using self-compassion mantras, and adopting the mindset of treating oneself as one would treat a friend (Neff and Germer, 2013).

Regarding the self-compassionate attitudes, the Compassion-Focused Therapy (Gilbert, 2010, 2015; Gilbert and Choden, 2014), suggests that patients with high self-compassion tend to present better mental health (Neff, 2003b; Neff et al., 2005, 2007; Lantyer et al., 2016; Nonnenmacher and Pureza, 2019; Van Niekerk et al., 2022), less pain catastrophizing (Pulvers and Hood, 2013; Hanssen et al., 2014), and suffering compared to patients with less self-compassion (Lantyer et al., 2016; Nonnenmacher and Pureza, 2019; Van Niekerk et al., 2022). In addition, self-compassion is associated with mechanisms that regulate pain, such as heart rate variability, the oxytocin and endorphin regulation systems (Lanzaro et al., 2021).

Self-compassion also works as emotion regulation strategy that can collaborate to the decrease of subjective negative states (Neff, 2003a,b; Neff et al., 2005, 2007; Cunha et al., 2013). Therefore, self-compassion is benefical in managing of chronic pain (Finan and Garland, 2015; Ong et al., 2015; Peters et al., 2017; Torrijos-Zarcero et al., 2021), including chronic low back pain (Cherkin et al., 2016; Michalsen et al., 2016; Zgierska et al., 2016; Reiner et al., 2019; Polaski et al., 2021). However, while more conscious attitudes help in the management of chronic pain, fear and emotional avoidance can harm mental health and treatment (Gilbert et al., 2014). The practice of conscious awareness through mindfulness is fundamental to ease suffering through self-compassion (Gilbert and Choden, 2014).

A prior systematic review highlighted the health benefits of self-compassion-related interventions among individuals with chronic physical health problems (Kilic et al., 2021). Compiled evidence suggests that self-compassion is associated with a decrease in physical pain, psychological distress, and improved parameters of positive mental health, such as positive affect (Kilic et al., 2021). Despite these studies, to the best of our knowledge, only one previous systematic review has evaluated the effectiveness of self-compassion-based interventions in chronic pain (Lanzaro et al., 2021). However, it selected articles from up to 2020, considering observational design articles.

Using the acronym PICO (population, intervention, comparator, and outcome), we formulated the following research question that guided the systematic review: what are the benefits of self-compassion interventions on the physical and mental health of adults with chronic low back pain? Adults refers to population, self-compassion, intervention, pain, and mental health to outcomes. Comparators were the studies’ own control groups.

## 2. Methods

### 2.1. Databases and search strategy

We conducted a systematic literature review according to Preferred Reporting Items for Systematic Reviews and Meta-Analyses (PRISMA) guidelines (Moher et al., 2009; Galvão et al., 2015; Page et al., 2021). The review protocol was registered in The International Prospective Register of Systematic Reviews (PROSPERO—Free et al., 2010) and is accessible under the ID number CRD42022376341. Since there are no guidelines on how databases should be chosen when conducting a systematic review, we chose them with the assistance of a librarian with full technical knowledge of the databases PubMed, LILACS, SciELO, PePSIC, PsycINFO, Embase, Scopus, Web of Science, and Cochrane. Table 1 presents the search terms used in combination with Boolean search methods. The terms are based on the Health Science Descriptors (DeCS/MeSH). All the searches were performed between November and December 2022. The language filter used in databases was English, Spanish, and Brazilian Portuguese (Brazil), no restrictions concerning publication date were applied.

**Table 1:** Search strategies.

Database	Search strategy
LILACS and PePSIC	(mh:(Self-Compassion) OR tw:(Autoperdão OR Autoperdón)) AND (mh:(Low Back Pain) OR tw:(Lumbago OR Lumbalgia OR lombalgia))
SciELO	subject:(Self-Compassion OR Autoperdão OR Autoperdón) AND subject:(Low Back Pain OR Lumbago OR Lumbalgia OR lombalgia)
Embase	(“Self Compassion”/exp. OR “Self-Compassion”:ti,ab,kw OR “Self-Forgiveness”:ti,ab,kw OR “Self Forgiveness”:ti,ab,kw) AND (“Low Back Pain”/exp. OR “Low Back Pain*”:ti,ab,kw OR “Low Back Ache”:ti,ab,kw OR “Low Backache”:ti,ab,kw OR “Lower Back Pain*”:ti,ab,kw OR Lumbago:ti,ab,kw OR “Mechanical Low Back Pain*”:ti,ab,kw OR “Postural Low Back Pain*”:ti,ab,kw OR “Recurrent Low Back Pain*”:ti,ab,kw)
Scopus	TITLE-ABS-KEY(“Self-Compassion” OR “Self Compassion” OR “Self-Forgiveness” OR “Self Forgiveness”) AND TITLE-ABS-KEY(“Low Back Pain” OR “Low Back Pain*” OR “Low Back Ache” OR “Low Backache” OR “Lower Back Pain*” OR “Lumbago” OR “Mechanical Low Back Pain*” OR “Postural Low Back Pain*"OR “Recurrent Low Back Pain*”)
Web of Science	TS = (“Self-Compassion” OR “Self Compassion” OR “Self-Forgiveness” OR “Self Forgiveness”) AND TS = (“Low Back Pain” OR “Low Back Pain*” OR “Low Back Ache” OR “Low Backache” OR “Lower Back Pain*” OR “Lumbago” OR “Mechanical Low Back Pain*” OR “Postural Low Back Pain*"OR “Recurrent Low Back Pain*”)
PsycINFO	((IndexTermsFilt: (“Self-Compassion”)) OR (Keywords: (“Self Compassion”) OR Keywords: (“Self-Forgiveness”) OR Keywords: (“Self Forgiveness”)) OR (abstract: (“Self Compassion”) OR abstract: (“Self-Forgiveness”) OR abstract: (“Self Forgiveness”)) OR (title: (“Self Compassion”) OR title: (“Self-Forgiveness”) OR title: (“Self Forgiveness”))) AND ((Keywords: (“Low Back Pain*”) OR Keywords: (“Low Back Ache”) OR Keywords: (“Low Backache”) OR Keywords: (“Lower Back Pain*”) OR Keywords: (“Lumbago”) OR Keywords: (“Mechanical Low Back Pain*”) OR Keywords: (“Postural Low Back Pain*”) OR Keywords: (“Recurrent Low Back Pain*”)) OR (title: (“Low Back Pain*”) OR title: (“Low Back Ache”) OR title: (“Low Backache”) OR title: (“Lower Back Pain*”) OR title: (“Lumbago”) OR title: (“Mechanical Low Back Pain*”) OR title: (“Postural Low Back Pain*”) OR title: (“Recurrent Low Back Pain*”)) OR (abstract: (“Low Back Pain*”) OR abstract: (“Low Back Ache”) OR abstract: (“Low Backache”) OR abstract: (“Lower Back Pain*”) OR abstract: (“Lumbago”) OR abstract: (“Mechanical Low Back Pain*”) OR abstract: (“Postural Low Back Pain*”) OR abstract: (“Recurrent Low Back Pain*”)))
Cochrane Trials	ID Search Hits
	#1 MeSH descriptor: [Self-Compassion] explode all trees 12
	#2 “Self Compassion” OR “Self-Forgiveness” OR “Self Forgiveness” 870
	#3 #1 OR #2870
	#4 MeSH descriptor: [Low Back Pain] explode all trees 4,577
	#5 “Low Back Pain*” OR “Low Back Ache” OR “Low Backache” OR “Lower Back Pain*” OR “Lumbago” OR “Mechanical Low Back Pain*” OR “Postural Low Back Pain*"OR “Recurrent Low Back Pain*” 12,840
	#6 #4 OR #512840
	#7 #3 AND #6 3
PubMed	(Self-Compassion[mh] OR Self Compassion[tiab] OR Self-Forgiveness[tiab] OR Self Forgiveness[tiab])
	AND
	(Low Back Pain[mh] OR Low Back Pain*[tiab] OR Low Back Ache[tiab] OR)
	Low Backache[tiab] OR Lower Back Pain*[tiab] OR Lumbago[tiab] OR
	(Mechanical Low Back Pain*[tiab] OR Postural Low Back Pain*[tiab] OR Recurrent Low Back Pain*[tiab])

### 2.2. Eligibility criteria

The inclusion criteria used to select papers were: randomized, longitudinal clinical trial studies that used the self-compassion construct in association with low back pain in adult patients. The exclusion criteria were: gray literature data, such as book chapters, dissertations, theses, review studies, abstracts of scientific events, and incomplete or unpublished studies, in addition to studies that addressed people with physical disabilities, cancer pain, fibromyalgia, rheumatic diseases, spinal fractures, individuals who had less than 5 years of schooling, less than 3 months of low back pain, and pregnant women.

Publications retrieved from databases were imported to the Rayyan (Ouzzani et al., 2016). Two independent reviewers analyzed the titles and abstracts of the articles according to the eligibility criteria. All studies that met the inclusion criteria were pre-selected for full-text reading and data were extracted from the included papers according to relevance.

### 2.3. Analysis of studies risk of bias and effect size

Studies risk of bias was assessed using The Risk of Bias 2 (RoB 2; Sterne et al., 2019). RoB 2 assesses the risk of bias across five domains: randomization process (D1), deviations from intended interventions (D2), missing outcome data (D3), measurement of outcomes (D4), and selection of reported results (D5), in addition to providing an overall assessment. Each of these domains, as well as the overall result, can be categorized as either low risk of bias, some concerns, or high risk of bias.

The interventions efficacy was analyzed based on Cohen *d* statistic reported in the studies. Two of the included articles did not present this measure as a result. In these cases, the Cohen *d* was calculated based on other reported statistics (e.g., Mean, standard deviation, and standard error) as suggested by the Cochrane manual (Higgins et al., 2022).

## 3. Results

**Figure 1:**
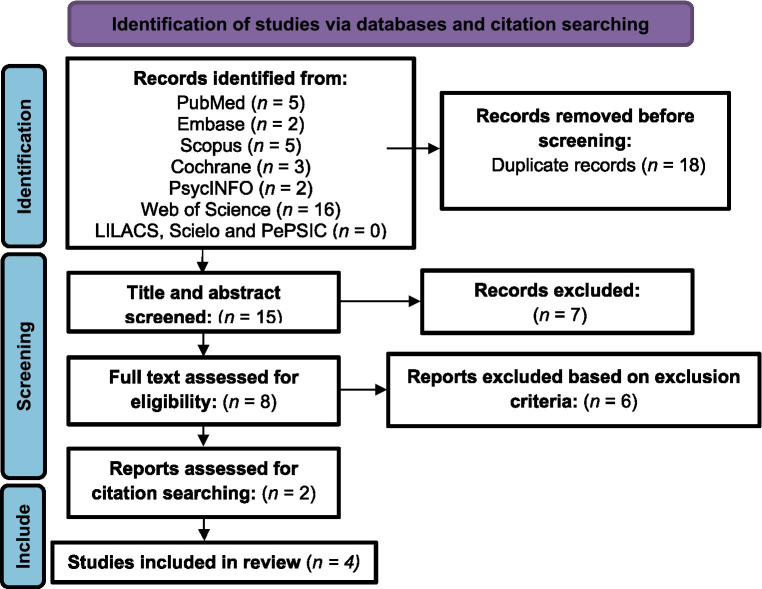
Search and identification process for included studies.

The initial search identified 33 studies, after duplicates were removed 15 records remained for title and abstract screening phase. In this phase, eight papers were selected for full text review. Six studies were excluded during the full text review based on the inclusion/exclusion criteria. After full text reading, a manual search was performed in the reference lists of the two remaining studies. Two more articles were included after reading the reference lists. Therefore, four articles were included in this systematic review. Of these, two were extracted from the PubMed database and two by searching the references lists. The Figure 1 show the steps of studies selection according with the PRISMA flowchart (Page et al., 2021).

Through the analysis of the included studies, it was possible to evaluate the effects and the relationship between self-compassion, low back pain, and associated biopsychosocial aspects. Table 2 shows the main publication characteristics of the four included studies, their samples, study design, intervention types, instruments, and results.

**Table 2:** Studies and sample characteristics.

Reference	Instrument	Sample	Study Design/Intervention Duration	Intervention Name/Techniques/Trained Instructors	Results
Berry et al. (2020)	MAIA	*n =* 20, Ma = 40.15 (S*D =* 12.56), 65% women, United States	Longitudinal clinical trial (2 weeks: 6 and 4 h). 1 week follow-up	Self-Compassion Training/Self-Compassion Psychoeducation and Loving-Kindness Meditation/Yes	Reduction in intensity (PROMIS) *d =* − 0.55 (*p =* 0.001) and low back pain disability (RMQ) *d =* − 0.63 (*p =* 0.001).
	PCS				
	PROMIS				
	RMQ				Increased self-compassion trait (SCS) *d =* 0.44 (*p =* 0.020) and Interoceptive awareness (MAIA) *d =* 0.46 (*p =* 0.04)
	SCS				
	CEQ				
Zheng et al. (2022)	NRS	*n =* 37, Ma = 35.2 (S*D =* 11.1), 75.7% women, China	RCT (4 weeks: 2 h). 16 weeks follow-up	Self-Compassion Training with Core Stability Exercise/Self-Compassion Psychoeducation and Loving-Kindness Meditation/Yes	Anxiety reduction (GAD-7) *d* = − 0,47 (*p* = 0.030). Although there was no significant difference, participants quickly improved pain disability, intensity, and catastrophizing
	GAD-7				
	PHQ-9				
	PCS				
	PSEQ				
	RMQ				
Chapin et al. (2014)	PROMIS	*n =* 12, Ma = 48.33 (S*D =* 10.80), 83.3% women, United States	RCT (9 weeks: 2 h). 9 weeks follow-up	Compassion Cultivanting Training/Self-Compassion Psychoeducation and Loving-Kindness Meditation/Yes	Reduction in pain intensity (BPI) *d* = − 0.82 (*p* = 0.003) and anger (PROMIS) *d* = − 0.68 (*p* = 0.01).
	BPI				Increase in pain acceptance *d* = 0.93 (*p* = 0.01)
	CPAQ				
Carson et al. (2005)	MPQ	*n =* 43, Ma = 51.1 (S*D =* not reported), 61% women, United States	RCT (8 weeks: 90 min). 3 months follow-up	Loving-Kindness Meditation Program/Self-Compassion Psychoeducation and Loving-Kindness Meditation/Yes	Reduction in pain intensity *d* = − 0.42 (*p* = 0.03), usual pain *d* = − 0.42 (*p =* 0.04) and psychological aspects (i.e., psychological distress, anxiety, anger, and tension) *d* = − 0.51
	BPI				
	STAXI-II				
	BSI				

MAIA, Multidimensional assessment of interoceptive awareness; PCS, Pain catastrophizing scale; PROMIS, Patient-reported outcomes measurement information system; RMQ, Roland-Morris disability questionnaire; SCS, Self-compassion scale; CEQ, Credibility/Expectancy questionnaire; NRS, Numerical rating scale; GAD-7, Generalized anxiety disorder scale seven item version; PHQ-9, Patient health questionnaire; PSEQ, Pain self-efficacy questionnaire; BPI, Brief pain inventory; CPAQ, Chronic pain acceptance questionnaire; MPQ, McGill pain questionnaire; STAXI-II, State–trait anger expression inventory; BSI, Brief symptom inventory; SD, Standard deviation; Ma, Mean age; RCT, Randomized clinical trial; d, Cohen’s effect size; *p* value, 95% significance level.

The articles were published between 2005 and 2022, and all assessed low back pain. The measurement instruments used were: Brief Pain Inventory (BPI—Daut et al., 1983), Patient-Reported Outcomes Measurement Information System (PROMIS—Craig et al., 2014), Pain Catastrophizing Scale (PCS—Sullivan et al., 1995), Multidimensional Assessment of Interoceptive Awareness (MAIA—Mehling et al., 2012), Roland-Morris Low Back Pain and Disability Questionnaire (RMQ—Roland and Morris, 1983), Self-Compassion Scale (SCS—Neff, 2003b), Credibility/Expectancy Questionnaire (CEQ—Devilly and Borkovec, 2000), Numerical Rating Scale (NRS—Herr et al., 2004), Generalized Anxiety Disorder Seven Item Version (GAD-7—Zhang et al., 2021), Patient Health Questionnaire (PHQ-9—Wang et al., 2014), Pain Self-Efficacy Questionnaire (PSEQ—Yang et al., 2019), Chronic Pain Acceptance Questionnaire (McCracken et al., 2004), McGill Pain Questionnaire (MPQ—Melzack, 1975), State-Trait Anger Expression Inventory (STAXI-II—Spielberger, 1999), and Brief Symptom Inventory (BSI—Derogatis and Melisaratos, 1983).

Regarding the studies design, three studies were randomized clinical trials (RCTs) and one was a longitudinal clinical trial. The population was predominantly adult women. In two of the four studies, participants had no experience with meditation. During studies interventions, all participants experienced some type of self-compassion meditation intervention. The interventions lasted between 2 and 9 weeks, with sessions ranging from one and a half to 6 h and involved self-compassion training through loving-kindness meditation in the four studies.

Self-compassion-related interventions exhibited efficacy in improving both mental health and pain parameters among adults with CLBP. In all the studies, statistically significant improvements were observed in mental health parameters, including anxiety, self-compassion, and anger. Regarding pain, all four interventions successfully mitigated its intensity, with three studies yielding statistically significant outcomes.

**Figure 2:**
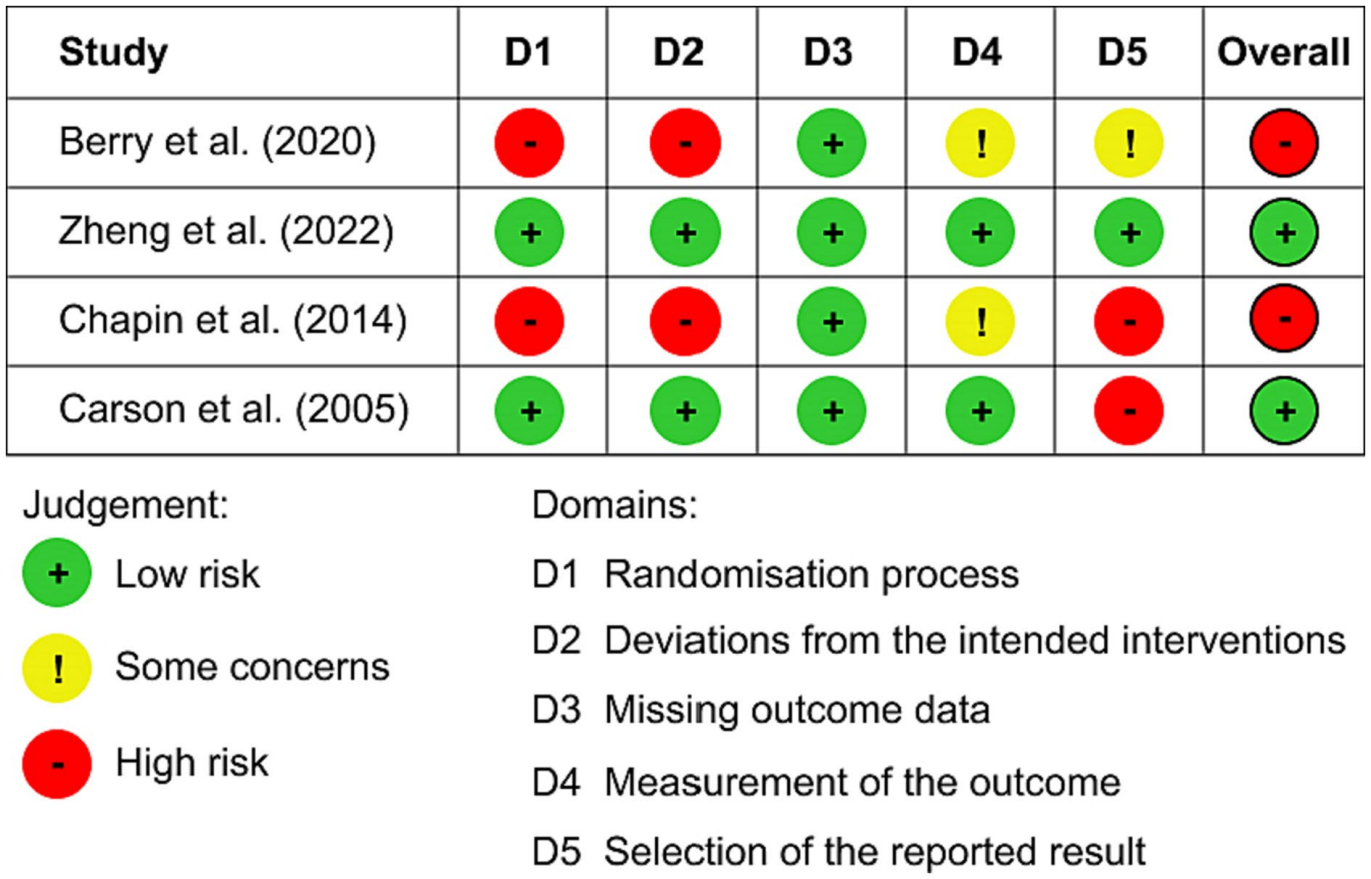
Risk of bias assessment.

Lastly, a summary of the possible biases in the selected studies is presented in Figure 2. Two studies exhibited a low risk of bias (Carson et al., 2005; Zheng et al., 2022), while two studies exhibited a high risk of bias (Chapin et al., 2014; Berry et al., 2020). The primary contributing factors to the high risk of bias were deficiencies in the participant randomization process (D1) and the application of measurement procedures (D4). Missing outcome data (D3) was the sole domain in which all studies demonstrated equally satisfactory performance.

## 4. Discussion

The present study aimed to assess the benefits of self-compassion-related interventions on biopsychosocial outcomes in adults dealing with CLBP. While previous systematic reviews have examined the relationship between self-compassion, pain, and mental health (Kilic et al., 2021; Lanzaro et al., 2021), to our knowledge, our systematic review is the first to exclusively focus on individuals with CLBP. A notable strength of this study lies in its exclusive inclusion of RCTs and longitudinal clinical trials, designed to provide more robust evidence regarding the potential of self-compassion-related interventions. In general, the findings suggest that such interventions hold promise for enhancing the mental health and decrease pain of adults with CLBP.

A statistically significant improvement in pain intensity was observed in three of the four studies. The effect sizes suggest that this improvement was of medium magnitude in the studies by Berry et al. (2020) and Carson et al. (2005), and of large magnitude in the study by Chapin et al. (2014) (Field, 2017). These results align with evidence from previous systematic reviews that have assessed the benefits of self-compassion interventions in chronic pain across various patient populations (Misurya et al., 2020; Kilic et al., 2021; Lanzaro et al., 2021). A possible explanation is that the practice of self-compassion activates brain regions associated with pain relief, in addition to releasing neurotransmitters that can also mitigate its effects (Lanzaro et al., 2021). However, it is important to note that the precise mechanism linking pain relief and self-compassion still requires further investigation for a more comprehensive understanding. Cultivating self-compassion also helps improve self-care and disease management, as more self-compassionate individuals start to treat themselves with more care and kindness (Misurya et al., 2020).

Regarding mental health, the results also highlighted the benefits of the self-compassion-related interventions. All interventions demonstrated improvement in at least one mental health parameter. The effect sizes suggest a moderate decrease in anxiety scores in the study conducted by Zheng et al. (2022), a moderate increase in self-compassion scores as shown by Berry et al. (2020), a moderate decrease in anger scores as observed in the study of Chapin et al. (2014), and a moderate decrease in psychological aspects (which encompasses emotional distress, anxiety, and anger scores) reported by Carson et al. (2005). These findings affirm the potential of self-compassion-related interventions in enhancing mental health and are corroborated by other evidence. Previous studies suggested that self-compassion may contribute to the reduction of anger, helplessness, catastrophizing, anxiety, fear, increased acceptance, and changes pain beliefs in individuals with low back pain (Sirois et al., 2015; Torrijos-Zarcero et al., 2021; Ashar et al., 2022). Furthermore, it is through the awareness of suffering gained through mindfulness (one of the components of self-compassion), that relief can come to the individual (Gilbert and Choden, 2014).

The self-compassion training through loving-kindness meditation (Neff, 2003a; Neff and Germer, 2013) and the self-compassion psychoeducation are the main intervention analyzed in this review. Self-compassion training is a favorable resource in unpleasant situations and can improve chronic pain through embracing and accepting suffering (Carvalho et al., 2018). The psychoeducation in self-compassion is based on cognitive therapy (American Psychiatric Association, 2013), and guides the individual to coping with chronic pain through the acceptance of one’s own feelings (Curtis and Pirie, 2018). From this perspective and according to the findings, psychological factors can complement non-pharmacological treatments in patients with low back pain (Smit et al., 2023). It is noteworthy that the most common, psychological factors in the literature are conscious self-compassion (Torrijos-Zarcero et al., 2021), and acceptance of chronic pain through meditation-based interventions (Garland et al., 2017; Dindo et al., 2018).

The self-compassion-related interventions were distributed between 2 and 9 weeks, with sessions lasting from one and a half to 6 h and involved the training of self-compassion through loving-kindness meditation in the four analyzed studies. This finding is supported by recent research that used meditation-based therapies for pain intensity reduction and observe treatment benefits after between 4 and 8 weeks (Cherkin et al., 2016; Michalsen et al., 2016; Zgierska et al., 2016; Reiner et al., 2019; Polaski et al., 2021). However, as with the results of this review, the literature suggests that individuals who have practiced meditation show better levels of well-being and self-compassion (Weiss et al., 2016; Camillo et al., 2022). Therefore, regardless of the degree of exposure, self-compassion contributes to mental health, psychological and physical well-being, and eases the suffering involved in low back pain (Almeida et al., 2021; Camillo et al., 2022).

On the other hand, owing to the limited number of studies and the considerable diversity among the study populations, it is challenging to definitively determine which intervention proved to be superior. Nevertheless, the study conducted by Chapin et al. (2014) demonstrated the most substantial effect size concerning chronic pain intensity. One plausible hypothesis is the intervention’s duration of 9 weeks, which was the lengthiest among all the included studies. Existing evidence suggests that intervention length may correlate with effect size in health interventions (Wakelin et al., 2021).

All the results found in this review are corroborated by the theoretical model on which it is based. Especially the reduction in pain intensity after interventions with meditation-based therapies. Therefore, meditation-based interventions, especially self-compassion meditation with psychoeducation in self-compassion, consider the context of the individual and are favorable non-pharmacological treatment options for low back pain (Camillo et al., 2022; Lin et al., 2022).

In comparison to the systematic review conducted by Lanzaro et al. (2021), this study presents some differences. Firstly, it is important to emphasize the inclusion of two newly published articles from 2020 to 2022. Additionally, a distinct feature of the present review lies in its deliberate exclusion of observational studies. This criterion enhances the precision of evidence regarding the causality between self-compassion interventions and biopsychosocial outcomes in individuals with CLBP.

### 4.1. Limitations

All studies employed meditation as the primary technique, with a particular focus on loving-kindness meditation. Loving-kindness meditation involves a specific type of breathing exercise that simultaneously encompasses the experience of self-kindness and the practice of mindfulness itself. Consequently, it may partially overlap with conventional mindfulness techniques. Therefore, a plausible hypothesis is that the observed results may stem from the mindfulness experience itself. On the other hand, it is crucial to emphasize that during the meditation practice, individuals are encouraged to extend self-kindness to themselves, which fundamentally distinguishes it from other mindfulness techniques. The overlapping nature of these constructs had been previously noted in a prior review (Wakelin et al., 2021).

Still regarding self-compassion techniques, none of the four studies presented in detail the names of the techniques used, with the exception of loving-kindness meditation and psychoeducation. Therefore, it is not possible to be sure which mechanism may have effectively contributed to the reduction in pain and improved mental health parameters. It is important that future studies test other resources of self-compassion-related interventions, such as the self-compassion letter, imagination of the compassionate self, self-compassion mantra, gratitude chart, and other widely used techniques (Neff and Germer, 2013).

Only one study assessed self-compassion as an outcome, representing a limitation. The remaining three studies should have assessed whether there were enhancements in self-compassion, with the intention of gaining a deeper insight into the connection between the suggested intervention and outcomes related to pain and mental health. It is imperative that forthcoming studies investigate whether there is an amelioration in self-compassion, as this would provide greater clarity regarding the key elements of the intervention. Additionally, the self-compassion scale enables us to evaluate which of its components were most influenced by the intervention, such as self-compassion, common humanity, or mindfulness (Neff et al., 2019).

The limited number of articles represents a constraint in this systematic review, hindering the generalization of results. Moreover, the studies featured small sample sizes, primarily comprising female participants from the United States and China. It is plausible that the language restriction to English, Spanish, and Portuguese may have resulted in the omission of potentially relevant articles. Conversely, it is crucial to underscore that one of the inclusion criteria was exclusively RCTs, which are the gold standard for establishing causal relationships (Hariton and Locascio, 2018), and longitudinal clinical trials. However, this stringent criterion may have restricted the retrieval of articles.

Finally, it is important to highlight that only two studies exhibited an overall low risk of bias as determined by the RoB 2 assessment (Carson et al., 2005; Zheng et al., 2022). Consequently, one should approach the results with caution, given that the remaining two studies (Chapin et al., 2014; Berry et al., 2020) are associated with an overall high risk of bias. It is imperative that forthcoming studies adopt more rigorous methodologies, including randomization and blinding, and pre-publish their research protocols.

## 5. Conclusion

Overall, the analyzed data demonstrated that self-compassion-related interventions improve the biopsychosocial factors involved in chronic low back pain, as pain and mental health parameters. Average meditation time was positively associated with increased self-compassion and acceptance. It was also associated with significant reductions in low back pain and its related biopsychosocial aspects (e.g., pain intensity, pain-generated disability, anxiety, anger, tension, and quickly pain relief). Therefore, self-compassion may be favorable and contribute to the non-pharmacological psychotherapeutic treatment related to low back pain, as a complementary and safe approach for patients with this condition. We further observed that the prescription of self-compassion exercises (i.e., loving-kindness meditation) is reproducible with training alone. In general, participants who try the meditation treatments through self-compassion training may experience relief of low back pain intensity and disability more quickly than those who try conventional treatment. Thus, it is suggested the adoption of therapeutic interventions based on self-compassion in the care of the individual with chronic pain, both to reduce stress and to manage pain.

Furthermore, this review findings suggest that there are few studies that specifically address the relationship between self-compassion, low back pain, and associated biopsychosocial factors, being one of the main gaps in the literature. However, research has been increasing in this direction, and the inclusion of follow-up is needed for knowledge about how long the benefits of the self-compassion component practices last. Considering the limited number of clinical trials assessing the benefits of self-compassion interventions for individuals with chronic low back pain, we are optimistic that the positive evidence uncovered in this review will serve as an incentive for additional research. Expanding the scope of interventions will enable the development of more robust systematic reviews, including meta-analysis procedures, and facilitate broader generalizability through larger sample sizes.

## Data Availability Statement

The data analyzed in this study is subject to the following licenses/restrictions: The review dataset can be consulted from the KG upon reasonable request. Requests to access these datasets should be directed to kellenufcspa@gmail.com.

## Author contributions

KG: Conceptualization, Data curation, Formal analysis, Investigation, Methodology, Project administration, Resources, Software, Visualization, Writing – original draft, Writing – review & editing. PC: Conceptualization, Data curation, Formal analysis, Investigation, Methodology, Software, Supervision, Visualization, Writing – original draft, Writing – review & editing. BS: Data curation, Methodology, Resources, Visualization, Writing – original draft, Writing – review & editing. CR: Conceptualization, Data curation, Funding acquisition, Investigation, Methodology, Project administration, Resources, Supervision, Validation, Visualization, Writing – original draft, Writing – review & editing.
